# Cost-Effectiveness of Treatment for Canine Parasites in Remote Indigenous Communities

**DOI:** 10.1007/s10393-025-01718-w

**Published:** 2025-05-21

**Authors:** Cameron Raw, Anke Wiethoelter, Rebecca J. Traub, Virginia Wiseman, Caroline Watts

**Affiliations:** 1 Melbourne Veterinary School, Building 400, Corner Park Drive and Flemington Road, Parkville, VIC 3052 Australia; 2 Department of Infectious Diseases and Public Health, Jockey Club College of Veterinary Medicine and Life Sciences, City University of Hong Kong, Kowloon, Hong Kong; 3 Kirby Institute, University of New South Wales, Sydney, NSW Australia

**Keywords:** economic evaluation, aboriginal and Torres strait islander, hookworm, ivermectin, flumethrin, oxibendazole

## Abstract

Zoonotic canine parasites and the vector-borne diseases they may carry can cause high morbidity and mortality in dogs and people. Many remote Indigenous communities in northern Australia have numerous free-roaming dogs, tropical climates favouring parasite development, and limited access to veterinary care, which can promote high prevalence of zoonotic parasites. To successfully combat parasites, treatment programs are needed, which are effective in reducing parasite burden and prevalence as well as being cost-effective and feasible. We compared canine parasite treatments in a Torres Strait Islander community setting, including oxibendazole/praziquantel tablets (OXI), moxidectin/imidacloprid spot-on (MOX), off-label oral ivermectin (IVM), afoxolaner chews (AFO), and flumethrin/imidacloprid collars (FLU). Cost surveys estimated the total annual and per-dog cost of each program. Markov modelling determined the cost per dog free of infection for each program using a government payer perspective over six-month and four-year time horizons. The annual cost per dog treated was $54.53 for OXI, $95.44 for MOX, $22.85 for IVM, $219.79 for AFO and $133.95 for FLU. IVM was less costly and more effective than other treatments against hookworm. FLU dominated in ectoparasite treatment. Sensitivity analyses supported these results. This study contributes cost-effectiveness data to inform parasite treatment program policy with aims of significant reductions in zoonotic canine parasite prevalence and subsequent reductions in environmental contamination with infectious parasite stages.

**Supplementary Information:**

The online version contains supplementary material available at 10.1007/s10393-025-01718-w.

## Introduction

The warm and humid environments of northern Australia favour the development and spread of several species of internal and external parasites that impact the health of dogs (O’Donel Alexander 1984; Gordon et al. 2017). Many of these parasites are also responsible for well-recognised zoonotic diseases transmitted to humans (Smout et al. 2017a; Raw et al. 2022). Dogs are commonly free-roaming in remote Aboriginal and Torres Strait Islander communities in Australia and can live near humans or as fringe-dwelling wild dogs (Constable et al. 2010; Ma et al. 2020a; Bennett and Archer-Lean 2023). Their mobility, proximity to and interaction with other animal species including humans provide ample opportunity for disease exchange either directly or indirectly through contact with, or ingestion of contaminated soil, food, water or via bites from arthropod vectors such as ticks and fleas (Smout et al. 2017a).

Important examples of canine zoonoses in the Australian tropics are soil-transmitted helminths (STHs) including hookworms (*Ancylostoma* spp.) and threadworms (*Strongyloides* spp.) (Bradbury and Traub 2016; Traub et al. 2021; Raw et al. 2022). While ectoparasites such as fleas, ticks and mites are not known to complete their lifecycles on humans, even transient infestations caused by sharing bedding with an infested dog can lead to transmission of vector-borne diseases such as bartonellosis, flea-borne spotted fever or symptoms of hypersensitivity in the form of pruritis (Barrs et al. 2010; Teoh et al. 2016; Teoh et al. 2018). This pruritis, while seemingly innocuous, can be a precursor to secondary chronic skin disease, rheumatic fever or rheumatic heart disease (O’Donel Alexander 1984; Elliot et al. 2006).

Recent research indicates that known zoonotic STHs such as *Ancylostoma caninum* and *Strongyloides* spp. are endemic in dogs in remote communities, with high levels of flea and tick infestations also detected (Raw et al. 2022, 2024). Due to barriers of remoteness and limited funding, veterinary care may be sporadic, infrequent or non-existent in these communities, and the potential for continued parasite endemicity and zoonotic risk to humans is high (Bennett and Archer-Lean 2023). As such, cost-effective approaches towards the control and elimination of these parasites in dogs is critical as part of broader, culturally responsive animal health programs.

The control of parasites in remote Indigenous communities relies upon efficacious, effective and feasible treatments. The Australian Pesticides and Veterinary Medicines Authority (APVMA) registers antiparasitic treatments following evidence that the product meets safety, efficacy, trade and labelling criteria (Department of Agriculture Forestry and Fisheries 2014). Labelling criteria of registered treatments designate a specific use such as treating hookworm in dogs. Many antiparasitic treatments can be administered by owners, provided that the labelled use is adhered to. ParaGard® (Boehringer Ingelheim), Advocate® (Elanco), NexGard® (Boehringer Ingelheim) and Seresto® (Elanco) are labelled for the control and treatment of parasites in dogs and can be purchased and administered by owners. Products such as these may be more recently developed and covered by patents, increasing unit costs (Christie et al. 2013). Other treatments may be used in an off-label manner, though these decisions must be made and administered or overseen by a veterinarian, thereby incurring additional costs. Ivermectin is an off-label treatment in dogs and has been the mainstay of many veterinary or non-governmental organisations (NGOs) parasite treatment programs in remote Aboriginal and Torres Strait Islander communities for decades (Wilks and Williamson 1998; Bradbury and Corlette 2006; Burleigh et al. 2015). Evidence of ivermectin’s efficacy in this setting has recently been established (Raw et al. 2024), and as it is no longer covered by a patent, it is inexpensive to procure.

Failure to control parasite species may have drastically different consequences with varying zoonotic potential, life cycles and pathogenic effects. For instance, high prevalence of *A. caninum* in dogs may lead to greater environmental contamination with hookworm eggs and infective larvae and a higher risk of zoonotic infection. A realistic and worthwhile goal is therefore to reduce environmental contamination and animal burdens, thereby minimising zoonotic transmission as well as animal morbidity. While goals of reducing environmental and animal burden are similar for external parasites such as ticks and fleas, they have vastly different life cycles, modes of infestation and pathogenic effects (O’Donel Alexander 1984; Dantas-Torres 2010). Treatments may instead be targeted at killing immediately following bites or ideally repelling before bites occur to avoid vector-borne disease transmission. While complete community elimination of fleas, ticks or hookworms would be desirable, it is unlikely due to wild host species such as wild dogs, dingoes or feral cats harbouring these parasites and contributing to environmental burdens (Dantas-Torres 2010; Smout et al. 2017b; Clark et al. 2018).

Cost-effectiveness studies of human antiparasitic treatments have been performed in many countries throughout the world (Turner et al. 2015); however, cost-effective parasite control strategies in dogs are scarce. A scoping review on the economic evaluation of control strategies aimed at reducing cystic echinococcosis in humans highlighted the incompleteness of cost data and lack of detailed analysis or sensitivity analysis to inform this topic (Widdicombe et al. 2022). Cost-effectiveness measures of animal health programs can guide policy and funding decisions by local government or NGOs with potential enhancements in cost-effectiveness when considered within a One Health framework acknowledging the interconnectedness of human, animal and environmental health. One Health policy and programs also need to be culturally relevant to be most effective, recognising how culture informs interactions between people, animals and the environment. In remote Aboriginal and Torres Strait Islander communities, local culture may be vastly different from other countries or other regions in Australia. An example of this is the spiritual and cultural importance of dogs to many Aboriginal communities for whom dogs are part of their Dreaming (Smith and Litchfield 2009; Constable et al. 2010). As such, cultural interactions with and management of dogs needs to be considered in analyses and resulting policy decisions.

Current practice varies between communities and may involve labelled or off-label treatments, with choices based on provider access to treatments, veterinarian preferences or cost. Despite high prevalence of parasites, many remote communities do not have any current dog parasite treatment programs in place. Thus, this study aims to inform future treatment programs by determining the cost-effectiveness of three treatments for the zoonotic canine hookworm *A. caninum* and three ectoparasite treatments for fleas and ticks under field conditions in the Torres Strait Islands.

## Methods

### Overview

In this study, we compared the cost-effectiveness of three endoparasite treatments and three ectoparasite treatments administered according to labelled instructions or in an off-label manner. The perspective for this cost-effectiveness analysis was the local government payer, as animal health programs are funded by the local council in these Torres Strait Islander communities and are funded by government bodies in some other Aboriginal communities (Ma et al. 2020b). Two separate time horizons were examined: the first at six months to reflect the study period; and a longer time horizon of four years to examine the effect of real-world application of treatments on community parasite prevalence.

### Study Setting and Population

The location and study population have been described in detail elsewhere (Raw et al. 2024). Briefly, dogs on three remote tropical islands in the Torres Strait in Queensland, Australia, were included in this study. The Torres Strait spans an area of over 48,000 km^2^ with Papua New Guinea and the northernmost extent of Queensland as its northern and southern borders, respectively. Islands were selected based on recommendations from the Torres Strait Islands Regional Council regarding dog numbers. Community leadership and consultation were crucial to the design and completion of this research. Consultations were conducted with local Environmental Health Worker staff, followed by consultation with elders and elected council representatives of all island groups and approval of a formal research proposal. Environmental Health Workers were involved in all stages of field work, communicating with community members and providing treatments.

As many dogs as possible were enrolled on each island with owner consent, with a total of 175 dogs included in the field trial. Dog populations varied on each island in the region regarding numbers, the balance of sex, age group and whether dogs were desexed or entire, though these factors were not found to be significant regarding treatment efficacy (Raw et al. 2024).

### Interventions

All enrolled dogs on each island were assigned to the same treatment arm and given treatments for both internal and external parasites as described below. Treatments were selected to allow comparison of registered treatments administered according to labelled instructions versus off-label use. Registered treatments were selected based on availability of sufficient quantities, and off-label use of ivermectin was selected based on its current widespread use in remote community animal health programs (Wilks and Williamson 1998; Burleigh et al. 2015). For the treatment of *A. caninum*, dogs received three-monthly treatments of either: (i) oral tablets administered at 22.5 mg oxibendazole/5 mg praziquantel per kilogram bodyweight (ParaGard®, Boehringer Ingelheim) given according to labelled instructions (treatment group OXI); (ii) topical combination 1% moxidectin/10% imidacloprid (Advocate®, Elanco) applied according to labelled instructions at 0.1 ml per kilogram bodyweight (treatment group MOX); or (iii) off-label oral ivermectin (Bomectin®, Elanco) administered at 200 µg/kg in bread with flavoured paste (treatment group IVM). For the treatment of fleas and ticks, dogs received either (i) oral chews administered at 2.5 mg afoxolaner per kilogram bodyweight (NexGard®, Boehringer Ingelheim) administered monthly according to labelled instructions (treatment group AFO); (ii) a 10% imidacloprid/4.5% flumethrin polymer matrix collar (Seresto®, Elanco) applied according to labelled instructions (treatment group FLU); or (iii) off-label oral ivermectin treatment administered at 200 µg/kg (IVM). Treatment coverage was 95% based on efficacy study field data (Raw et al. 2024). This study was approved by the University of Melbourne Animal Ethics Committee (ID:10298).

### Cost Data Collection

Costs were estimated to conduct an ongoing treatment program assuming no associated research activities. Costs included capital costs of equipment for preparation of treatments and operational costs such as vehicle rental and fuel usage, purchase of parasite treatment, consumable transport and personnel costs including council and veterinary staff. Based on field data, approximately 14% of dogs in the FLU treatment group lost their collars each six-month period, and a replacement cost was included to account for additional collars used. All other treatments were administered topically or orally based on the weight of the dog and did not require replacement. Council environmental health worker staff costs were derived from publicly available council award rates and calculated based on an hourly rate inclusive of council administration costs (McLennan 2021). The time taken to administer treatments and the proportion of dogs in each weight group were based on observed dogs and treatment timing in this field trial. Vehicle usage costs per hour were derived from council fleet information inclusive of administration costs and were based on the time in use for the program. The costs of transporting parasite treatments from the supplier to the study islands were obtained from supplier shipping quotes and communication with council staff (E Gunn, personal communication, May 5, 2021). Parasite treatment costs were based on wholesale veterinary supplier prices (Provet 2021). Approximate tender costs for private veterinary clinics to provide services to the council region were obtained from council staff (E Gunn, personal communication, August 7, 2023). The overall tender cost of $135,000 is inclusive of veterinary staff wages, travel, and accommodation for veterinary staff to visit all communities across the Torres Strait Islands Regional Council area. These visits also included desexing and general animal health services; thus, only 15 min of the time spent in serviced communities per 100-dog cohort per cycle was included in this cost-effectiveness analysis, which accounts for the estimated veterinary time required to provide advice and oversight for ivermectin treatment. Total program costs per island were divided by the number of dogs treated to calculate a cost per dog treated. Prices are in June 2023 Australian dollars adjusted from 2021 figures from the time of the field trial using the Reserve Bank of Australia’s Inflation Calculator (Reserve Bank of Australia 2023). Cost inputs are presented in Table 1.

**Table 1:** Base Case Costs for Antiparasitic Treatment Interventions in Dogs

	ParaGard tablets (OXI)	Advocate spot-on (MOX)	Off-label ivermectin (IVM)	NexGard chews (AFO)	Seresto collars (FLU)^a^
5 kg dog treatment cost	$2.58	$17.92	$0.08	$12.83	$54.23
10 kg dog treatment cost	$4.37	$19.71	$0.16	$13.53	$54.23
20 kg dog treatment cost	$6.54	$19.71	$0.32	$13.53	$54.23
40 kg dog treatment cost	$13.08	$21.75	$0.63	$14.38	$54.23
Average 27.5 kg dog treatment cost	$9.20	$20.55	$0.43	$13.88	$54.23
Time to give treatment to each dog (hours)	0.05	0.033	0.017	0.05	0.055
Staff cost per dog treated^b^	$2.22	$1.46	$0.75	$2.22	$2.44
Time to prepare each treatment (hours)	–	–	0.004	–	–
Staff cost per dog treatment prepared^b^	–	–	$0.18	–	–
Cost of equipment to prepare treatment^c^	–	–	$0.49	–	–
Veterinary oversight time per dog treated (hours)	–	–	0.0025	–	–
Veterinary oversight cost^d^	–	–	$3.18	–	–
Vehicle hire cost per dog treated^e^	$1.09	$0.72	$0.37	$1.09	$1.20
Treatment transport per dog treated^f^	$1.13	$1.13	$1.13	$1.13	$1.13
Average cost per dog treated each treatment cycle	$13.63	$23.86	$5.71	$18.32	$66.98
Treatment cycle length (months)	3	3	3	1	6
Annual cost per dog	**$54.53**	**$95.44**	**$22.85**	**$219.79**	**$133.95**
Annual cost per 100-dog cohort	**$5,453.03**	**$9,544.49**	**$2,285.86**	**$21,979.20**	**$13,395.76**

a Same cost for collars for all sizes of dog.

b Staff salary cost based on hourly rate of $44.37.

c Equipment cost to prepare IVM treatments (needle, syringe, knife, cutting board) divided by 100-dog cohort.

d Cost to provide veterinary advice and oversight for administration of off-label treatments including travel, accommodation and support staff based on an hourly rate of $1271.20 and divided by the 100 dog cohort.

e Vehicle hire cost from fleet department based on hourly rate of $21.75 multiplied by the length of time to treat each dog.

f Transport for treatment for 100 dogs from warehouse to islands via local government office.

### Intervention Outcomes

Medium- to long-term effectiveness was modelled based on study results from a published field efficacy trial conducted in 2021, from which the following efficacy and prevalence data were drawn (Raw et al. 2024). Dogs were considered cured of hookworm infection if they were tested positive via quantitative PCR at baseline and were negative 7–11 days post-treatment. For ectoparasites, if dogs had fleas or ticks at baseline and were free of any observable fleas or ticks 7–11 days post-treatment, they were deemed cured. Efficacy against canine hookworms was 9% (95% CI 4.4 to 17.4) for OXI, 56.4% (95% CI 41 to 70.7) for MOX, and 89.7% (95% CI 73.6 to 96.4) for IVM. Efficacy against fleas was 100% (95% CI 88.6 to 100) for AFO, 100% (95% CI 75.8 to 100) for FLU and 0% (95% CI 0 to 19.4) for IVM. Efficacy against ticks was 67.3% (95% CI 53.8 to 78.5) for AFO, 80% (95% CI 58.4 to 91.9) for FLU and 4.3% (95% CI 0.8 to 21) for IVM.

Baseline prevalence for modelling effectiveness was the average of the baseline prevalence across all trial locations, which was 72.4% (95% CI 65.1 to 78.9) for *A. caninum*, 33% (95% CI 26.1 to 40.4) for fleas and 54.3% (95% CI 46.6 to 61.8) for ticks. To establish a dynamic model, a hookworm reinfection constant was calculated based on field trial data to account for the proportion of dogs reinfected during each three-month period. This constant was multiplied by the proportion of infected dogs in each community, which would determine the degree of environmental contamination with infective hookworm larvae. A reinfection constant was also applied to flea and tick models.

Effectiveness was measured as the proportion of dogs free of hookworms, fleas or ticks at each time horizon. A survival analysis for the prevalence of *A. caninum* infections in dogs for each treatment cycle was conducted with 95% confidence intervals of efficacy for each treatment.

Modelling was based on a population of 100 dogs per treatment group. Weights of dogs were estimated at the time of the field efficacy trial, of which 10% of enrolled dogs were 5 kg, 10% were 10 kg, 30% were 20 kg and 50% were 40 kg. The arithmetic mean dog weight was 27.5 kg, which was used to establish treatment costs for the dog population.

### Cost-Effectiveness Analysis

Calculated incremental cost-effectiveness ratios were defined as the cost per dog free of infection at each time horizon for each parasite of interest. A Markov model was created with cycle lengths of three months, considering different treatment intervals for each intervention (S1–S3). All modelling and analyses were conducted using TreeAge Pro Healthcare, v23 (TreeAge Software, Inc, Williamstown, Massachusetts, USA), with model outputs plotted in Microsoft Excel v 1908 (Microsoft Corporation, Redlands, California, USA).

### Sensitivity Analysis

Multiple one-way deterministic sensitivity analyses were conducted based on the four-year time horizon to examine the effect of differences in treatment cost, numbers of dogs, mean weights of dogs, vehicle and staffing costs, time taken to administer or prepare treatments and treatment efficacy. Upper and lower values of treatment efficacy were based on the 95% confidence intervals of each treatment in the previously described field trial (Raw et al. 2024). Likely upper and lower values of treatment costs were derived from variation in online retailer and wholesaler estimates, excluding bulk purchase discounts for the upper value. Ranges for treatment transport were derived from the least and most expensive shipping supplier quotes. The range tested for staff costs was based on variation in council staff award rates and for vehicle costs was based on daily rate estimates in hiring costs for shortest and longest time used from council fleet services. The likely values for numbers of dogs treated were based on field experience of the numbers of dogs seen in remote communities, while values for weights of dogs and the time needed to treat each dog were based on the ranges observed in this field study.

## Results

The mean annual cost to treat each 100-dog cohort was $5,453.03 for OXI, $9,544.49 for MOX, $2,285.86 for IVM, $21,979.20 for AFO and $13,395.76 for FLU. The total costs of treatment per dog as well as treatment of the 100-dog cohort are presented in Table 1. The contribution of treatment costs to total costs per cycle varied between treatment groups. Treatment costs accounted for 68% of total costs per cycle for OXI, 86% for MOX, 8% for IVM, 76% for AFO and 81% for FLU. Veterinary oversight costs accounted for 56% of IVM cost per cycle.

For the treatment of hookworm, IVM dominated MOX and OXI at both the six-month and four-year time horizons in terms of cost per dog free of infection. For the treatment of fleas and ticks, while IVM had a low cost per dog free of infection due to its very low overall cost, its effectiveness was poor compared to FLU and AFO. FLU dominated AFO at each time horizon for both flea and tick treatment. Markov cohort analyses are presented in Table 2. This table shows the cumulative costs and cost per dog free of infection for each parasite of concern at each time horizon.

**Table 2:** Cumulative Treatment Costs and Cost Per Dog Free of Infection at Each Time Horizon.

			Six-month time horizon			Four year time horizon		
Parasite	Dogs free of infection at baseline (/100)	Treatment	Dogs free of infection (/100)	Cumulative cost	Cost per dog free of infection	Dogs free of infection (/100)	Cumulative cost	Cost per dog free of infection
Hookworm	28	ParaGard tablets (OXI)	27	$2726.51	$100.98	28	$21,812.12	$779.00
		Advocate spot-on (MOX)	69	$4772.24	$69.16	99	$38,177.96	$385.64
		Off-label ivermectin (IVM)	89	$1185.80	$13.32	100	$9143.43	$91.43
Fleas	67	NexGard chews (AFO)	100	$10,989.60	$109.90	100	$87,916.80	$879.17
		Seresto collars (FLU)	100	$6697.88	$66.97	100	$53,583.05	$535.83
		Off-label ivermectin (IVM)	66	$1185.80	$17.97	59	$9143.43	$154.97
Ticks	46	NexGard chews (AFO)	99	$10,989.60	$111.01	100	$87,916.80	$879.17
		Seresto collars (FLU)	97	$6697.88	$69.05	100	$53,583.05	$535.83
		Off-label ivermectin (IVM)	49	$1185.80	$24.2	67	$9143.43	$136.47

**Figure 1:**
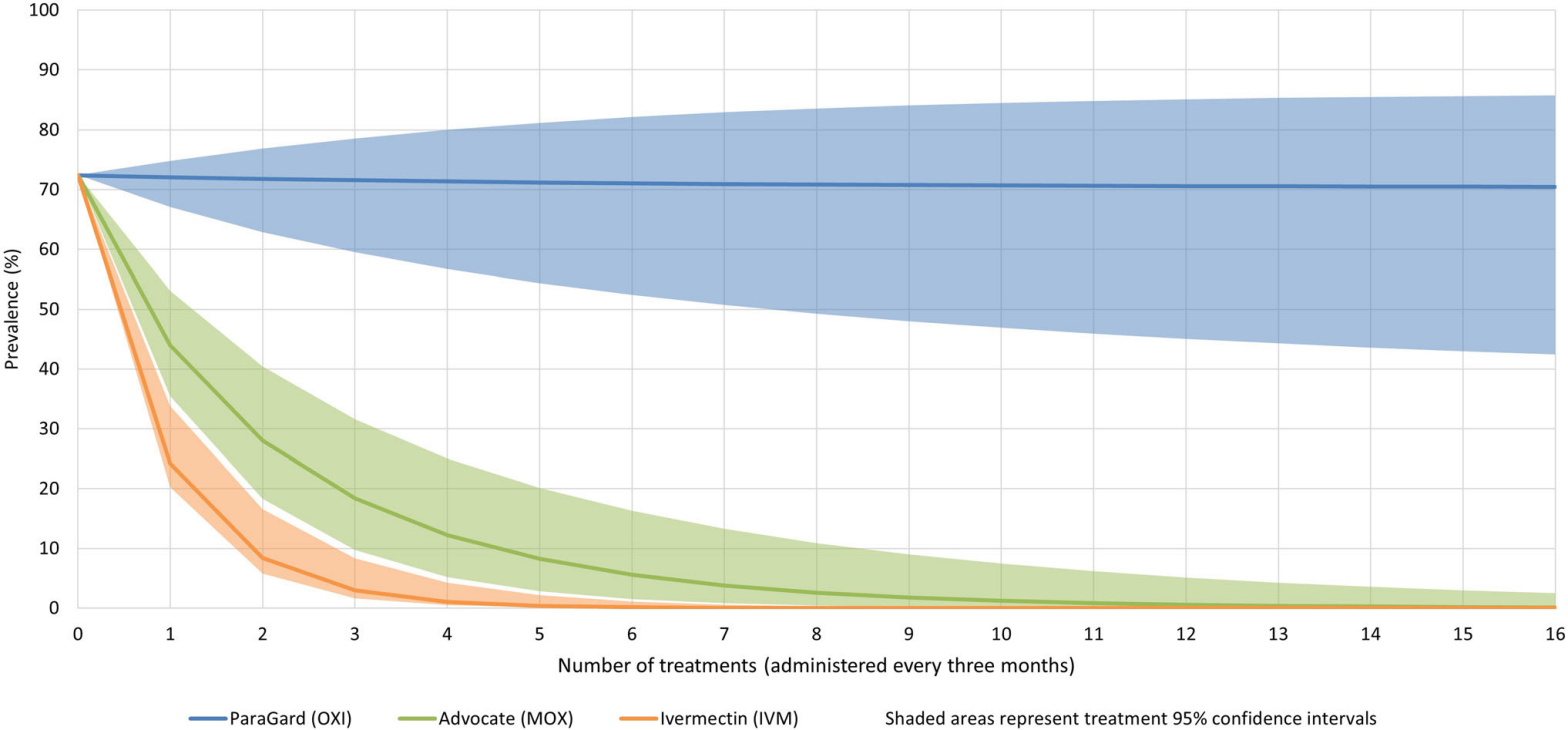
*Ancylostoma caninum* prevalence in dogs over time with treatment.

**Figure 2:**
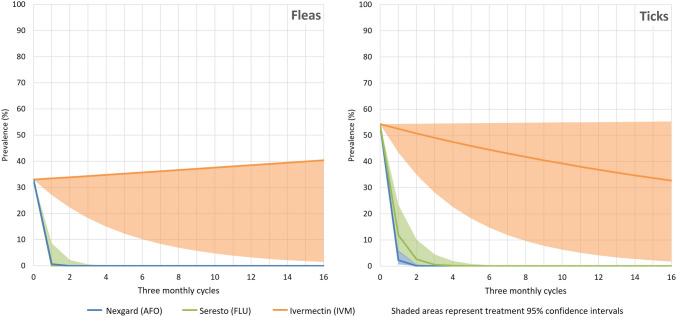
Flea and tick prevalence in dogs over time with treatment.

The reinfection constant for hookworm was calculated to be 0.25, while for ectoparasites it was 0.02. While rapid declines in hookworm prevalence were seen for MOX and IVM, the efficacy of OXI was insufficient to overcome reinfection rates in each treatment cycle. A survival curve of *A. caninum* prevalence in each treatment cycle is presented in Fig. 1, and survival curves for flea and ticks are presented in Fig. 2.

### Sensitivity Analysis

**Figure 3:**
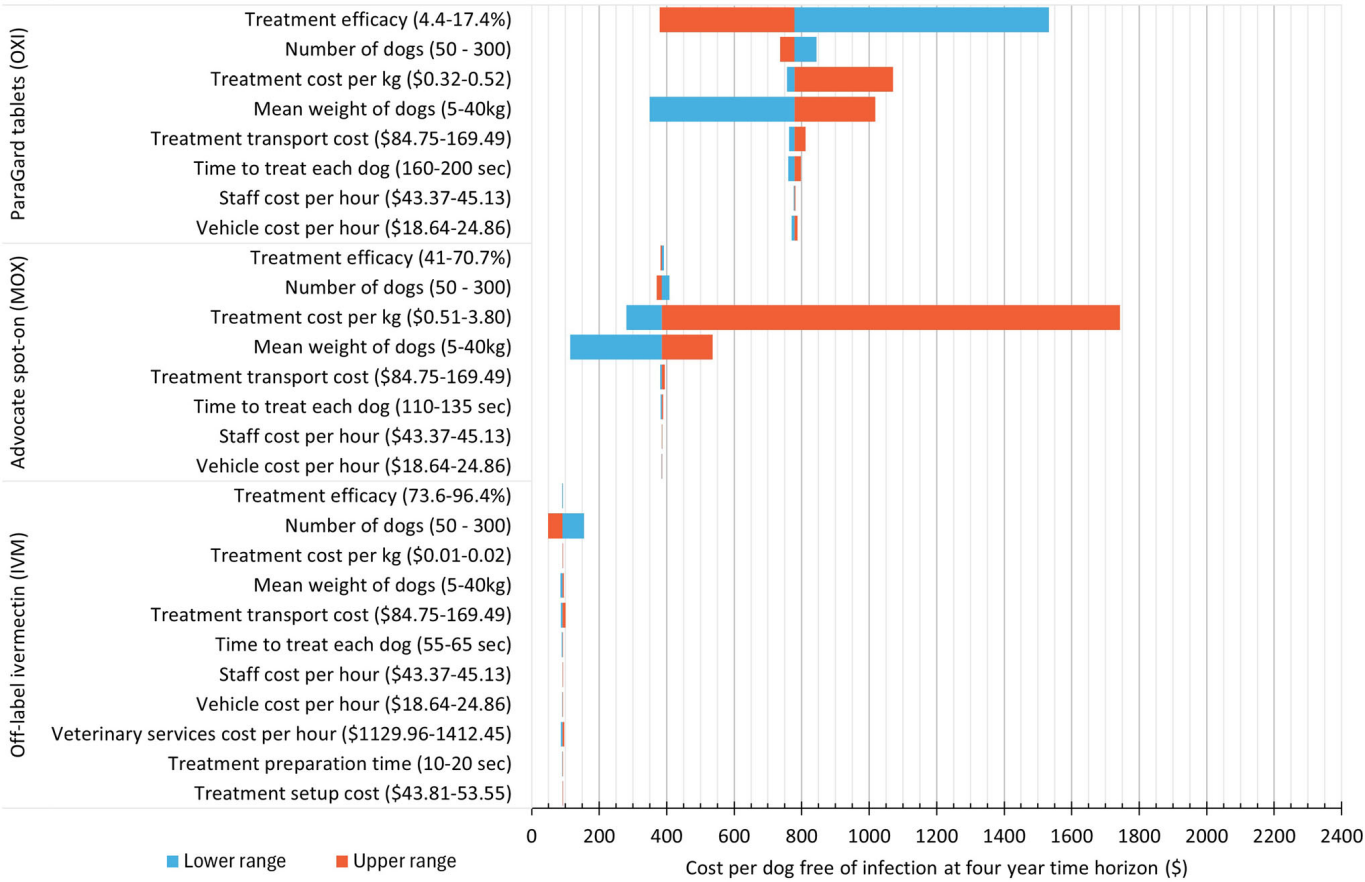
Tornado diagram of multiple one-way sensitivity analyses to examine the effect of variations in efficacy, cost, time and dog variables on the cost per dog free of *Ancylostoma caninum* infection at the four-year time horizon.

Multiple one-way deterministic sensitivity analyses revealed the same treatment dominance results as presented in Table 2, in which IVM consistently dominated both MOX and OXI in terms of cost per dog free of hookworm infection at the four-year time horizon regardless of variations in the cost or efficacy parameters of all three treatments. Similarly, MOX had lower cost per dog free of infection for all analyses in comparison with OXI except in the case of efficacy for which OXI dominated at its upper range. Increasing the number of dogs resulted in increased efficiency as reflected by a lower cost per dog free of infection for each treatment. Sensitivity analyses are presented in a tornado diagram in Fig. 3.

## Discussion

Owing to its low cost and high effectiveness, IVM was shown to be cost-effective compared to OXI and MOX, dominating in all modelled cost-effectiveness analyses for canine hookworm including sensitivity analyses. For the four-year time horizon as shown in Table 2, IVM resulted in 257% and 1% more dogs being free of infection at 42% and 24% of the cost of OXI and MOX, respectively. While IVM was less costly than AFO and FLU, it was ineffective against ticks and especially fleas. AFO and FLU were both highly effective against fleas and ticks, with FLU dominating AFO in primary and sensitivity analyses. Diminishing returns of the cost per dog free of infection were seen in all treatment arms over the two time horizons due to the movement of modelled cohorts towards elimination of infection. High treatment coverage was a strength in this study, though this cannot be relied upon in all community settings. It is worth noting that MOX in this study was administered at an off-label frequency of once every three months as opposed to the labelled monthly recommendation. On consultation, it was determined that monthly administration was not a feasible addition alongside the many duties of Environmental Health Worker staff. If monthly treatment was strictly adhered to, costs for MOX would triple across the modelled time horizons. Current literature for the effectiveness of moxidectin used at longer intervals is lacking, with evidence of its use against mites, heartworm and hookworm being at either monthly or shorter intervals (Castro et al. 2019; Schraven et al. 2021).

Care should be taken in comparing the overall costs of on- and off-label treatments, as the costs of veterinary oversight for off-label treatments can be considerable. In this model, 56% of the overall cost of ivermectin was due to veterinary costs. In the communities in this study, veterinary services were already being contracted for population control and general health care programs. A small amount of veterinary time was accounted for in this model to allow for the legally required veterinary oversight of off-label treatment as well as any training or discussions with animal health staff. Due to remoteness, communities often share veterinary services where a range of duties are completed including surgical or chemical neutering, parasite treatments and general health care (Wilks and Williamson 1998; Bradbury and Corlette 2006; Hiby et al. 2017). Efficiencies and economies of scale and scope may be achieved as numbers of dogs treated, numbers of communities visited and range of veterinary activities performed increase (Dias et al. 2015). Under these arrangements, it is feasible to include veterinary oversight for use of off-label treatments, but this may not be cost-effective if costs were not shared across communities due to very high travel and transport costs (Wilks 2000; Rural and Regional Affairs and Transport References Committee 2019).

Care should also be taken when considering the cost-effectiveness of treatments in isolation of other diseases. While ivermectin proved cost-effective for *A. caninum* infections, it was not cost-effective for ectoparasite infestations due to its lack of effectiveness. While ivermectin’s effectiveness against other important zoonotic helminths such as *Strongyloides stercoralis* is untested in dogs in this setting, it is reported to be effective in shelter dogs and humans in remote Indigenous communities (Kearns et al. 2017; Hays et al. 2017; Paradies et al. 2019). To optimise the use of staff time in parasite treatment programs, all potential internal and external parasite infections should be considered, especially those of zoonotic importance.

Treatment choice should be guided by priorities of the community and their desired outcomes. For fleas and ticks, prevention and repelling infestations are of primary importance due to the potential for vector-borne disease transmission from single bites (Otranto et al. 2008; Barrs et al. 2010; Teoh et al. 2018). For *A. caninum*, individual cure and reduction and elimination of community prevalence are meaningful aims. This is particularly relevant in a One Health framework given *A. caninum*’s zoonotic importance, the contribution to environmental contamination dependent on prevalence and host species range (Smout et al. 2017b; Raw et al. 2022). Modelling of human hookworm prevalence has revealed that driving overall prevalence below 2% via mass drug administration is likely to break transmission cycles resulting in community elimination and no further need for mass treatments (Truscott et al. 2017). Questions arise about the diagnostic sensitivity and specificity of tests used to determine when these thresholds have been reached and the cost of performing large numbers of tests required to detect infection in low prevalence settings (Lim et al. 2018). The alternative of ongoing mass treatment may involve lower costs compared to testing but carries a risk of anthelmintic resistance emergence (Bethony et al. 2006). Nonetheless, this study indicates that elimination of hookworm may be feasible in this setting with the use of ivermectin, as 2% prevalence was reached after only four treatment cycles. Ongoing treatment may be administered at longer intervals of six months or potentially ceased following confirmation of elimination.

Without similar studies available on the topic of cost-effectiveness of parasite treatment programs in dogs, it is challenging to draw comparisons to other settings. Most published analyses have examined cost-effectiveness of STH treatment strategies based on age group or target parasites, which has relevance in a broader One Health context but less relevance to the current study (Montresor et al. 2001; Turner et al. 2015). The results of this study are likely to be applicable to other Aboriginal and Torres Strait Islander communities in tropical and equatorial climatic zones in Australia with large populations of dogs. Such communities are present across the north of Queensland, Western Australia and the Northern Territory in which continued endemicity of *A. caninum* and *Strongyloides* spp. is evident (Raw et al. 2022). Communities with climates favourable to STH and ectoparasite development and limited access to veterinary care or parasite treatments can also be found throughout Oceania (Bradbury and Traub 2016; Page et al. 2016; Beknazarova et al. 2016; Traub et al. 2021). Similar regimens could also be cost-effective elsewhere for large populations of free-roaming dogs. Treatment, transport and staff costs may vary widely in non-Australian locations, and cultural and social factors determining human-animal interactions should be considered based on thorough community consultation. Using the methods within this study and substitution of locally relevant variables, these economic evaluations are likely to provide relevant cost-effectiveness outputs for parasite treatment programs in a range of settings globally.

The funder may also vary between locations and may include dog owners, government departments, or NGOs. While cost-effectiveness data may have meaning for the choice of parasite treatments by owners, it is the widespread, often multi-community council or NGO-operated programs with budgetary constraints that will benefit most from this information (Burleigh et al. 2015; Baker et al. 2018). Costs borne by owners may be limited to treatments, though costs borne by government may also extend to broad animal health or One Health programs. As such, the sustainability and ongoing resourcing of animal health programs in Indigenous communities are of great importance. Certainty in ongoing resourcing is likely to permit greater efficiencies in program planning, as well as in the establishment and strengthening of crucial cultural understandings and relationships. Efficiencies may be achieved even when different diseases are being targeted in different species within a One Health framework. In a study in Tanzania, an integrated health delivery platform conducted mass drug administration for STHs in humans and vaccination for rabies in dogs resulting in significant time savings and similar coverage when compared to each program delivered individually (Lankester et al. 2019). Additionally, it resulted in a 33% and 16% lower cost per deworming dose and rabies vaccination, respectively. Such research highlights an important area for further study in remote community settings, given that remoteness necessitates the efficient use of resources and time.

A limitation of this study is the uncertainty of model inputs such as the applied reinfection constant. While this was calculated from field trial data over a six-month period, it may not reflect the true rate of reinfection from environmental sources, particularly considering environmental variation between seasons. This is true for hookworms as well as fleas and ticks, which spend a greater proportion of their life cycle in the environment and may have a greater range of reservoir host species around communities, further adding to the complexity of real-world interactions (Dantas-Torres 2010). Modelling of flea and tick infection is also complicated by the mechanisms of action of the included treatments. Flumethrin/imidacloprid collars act as repellents, whereas afoxolaner chews act on the parasite after biting the host which makes measuring effectiveness challenging (Brianti et al. 2013; Shoop et al. 2014). Further studies in remote community settings are warranted to examine new parasiticides and other registered treatments, which may be administered by owners. Potential ecological impacts of any mass drug administration should also be considered with the One Health framework. Further study is also recommended in this area, particularly regarding the use of mass drug administration, to ensure that policy and practices remain sustainable.

## Conclusion

This study presents practical cost-effectiveness data for three endoparasite treatments and three ectoparasite treatments for dogs in remote northern Australian Aboriginal and Torres Strait Islander community settings. In these settings, reduction of environmental contamination with hookworm larvae may be possible by using ivermectin to treat infected dogs, and reduction of flea and tick bites in dogs may be feasible using combination flumethrin/imidacloprid collars. Practical and meaningful One Health outcomes are also possible in conjunction with regular anthelmintic treatments in people. Effective One Health programs involve community partnership and agency in health outcomes for animals, people and the environment.

## Supplementary Information

Below is the link to the electronic supplementary material.

Supplementary file1 (ZIP 27 kb)
